# Rare Association of Patent Foramen Ovale and Atrial Septal Aneurysm Leading to Branch Retinal Artery Occlusion in a Young Healthy Man

**DOI:** 10.7759/cureus.8994

**Published:** 2020-07-03

**Authors:** Phool Iqbal, Abdullah Shams, Mohammad Ali, Bassam Muthanna, Tanweer Hussain

**Affiliations:** 1 Internal Medicine, Hamad General Hospital, Doha, QAT; 2 Internal Medicine, CMH Lahore Medical and Dental College, Lahore, PAK; 3 Internal Medicine, Hamad Medical Corporation, Doha, QAT

**Keywords:** branched retinal artery, atrial septal aneurysm, healthy young man, patent foramen oval

## Abstract

Retinal artery occlusion (RAO) occurs in the elderly population above the age of 60 years due to carotid atherosclerosis as a consequence of long-standing hypertension, diabetes mellitus, smoking, and hyperlipidemia. It can also develop due to paradoxical emboli from patent foramen ovale (PFO), which can happen in a relatively younger population. Early diagnosis mandates prompt management; otherwise, it may lead to vision loss. We present a rare case of branch RAO (BRAO) in a healthy young gentleman with concurrent PFO and large atrial septal aneurysm, which has not been reported much in the literature.

Our patient presented with sudden left-sided blurriness of vision, which was diagnosed as BRAO on ophthalmoscope examination. Multidisciplinary teams were involved in reaching the underlying etiology of such a presentation in a young, healthy person. Urgent head CT with cerebral angiography and head MRI was unremarkable for any acute insult. The autoimmune screen and thrombophilia workup were unremarkable. After thorough investigations, a small PFO with a large atrial septal aneurysm was found to be correlating with his clinical picture.

We aim to highlight the importance of timely diagnosis and further management in such clinical scenarios, where permanent vision loss can compromise someone’s quality of life.

## Introduction

Patent foramen ovale (PFO) is a physiological opening present in fetal life between left and right atria and is pathological when it persists in adult life. It is present in 20-30% of the population and is found generally in 27% of the patients who have previously undergone routine autopsy [1]. However, in a majority of cases, it is not a harmful condition. It can act as a potential source of a paradoxical embolus from venous circulation through the left atrium and into the systemic circulation [2,3].

Central retinal artery occlusion (CRAO) is uncommon in young, healthy patients; however, if presents, it is mostly attributed to underlying PFO in 40% of the cases, leading to cryptogenic stroke in patients younger than 45-55 years of age [2,4]. It occurs mainly in the elderly population above 60 years of age and is a concern for 1/10,000 of the general population [5]. In the literature, most reported cases had shown PFO causing CRAO, with very few highlighting its relation with branch retinal artery occlusion (BRAO) [1,2,4,6]. Concomitant PFO and atrial aneurysm in young, healthy patients cause BRAO to create a more challenging clinical presentation in terms of rarity and management, thus making it a reportable case for further treatment guidance and follow-up [3,6].

We report the case of a patient who developed an acute episode of left eye vision dimness with blurring and was diagnosed as a case of BRAO due to PFO and atrial aneurysm.

## Case presentation

A 31-year-old young man without any prior medical condition presented acutely with left-sided decreased and blurred vision from two days. His symptoms started while he was watching a football match on TV. The patient described his visual disturbance similar to the appearance of a static TV with black and white dots. It was painless and occurred in the absence of trauma. There was no associated headache, nausea, vomiting, or any other neurological deficit. He was conscious oriented and was anxious about this episode. There was no history of previous such attacks. Further history of the systemic review was unremarkable.

Urgent ophthalmologist review and examination of both eyes revealed left macular edema, engorged vessels, bulging optic disc, and papillophlebitis consistent with occlusion of the superior-nasal branch of the retinal artery or BRAO.

**Figure 1:**
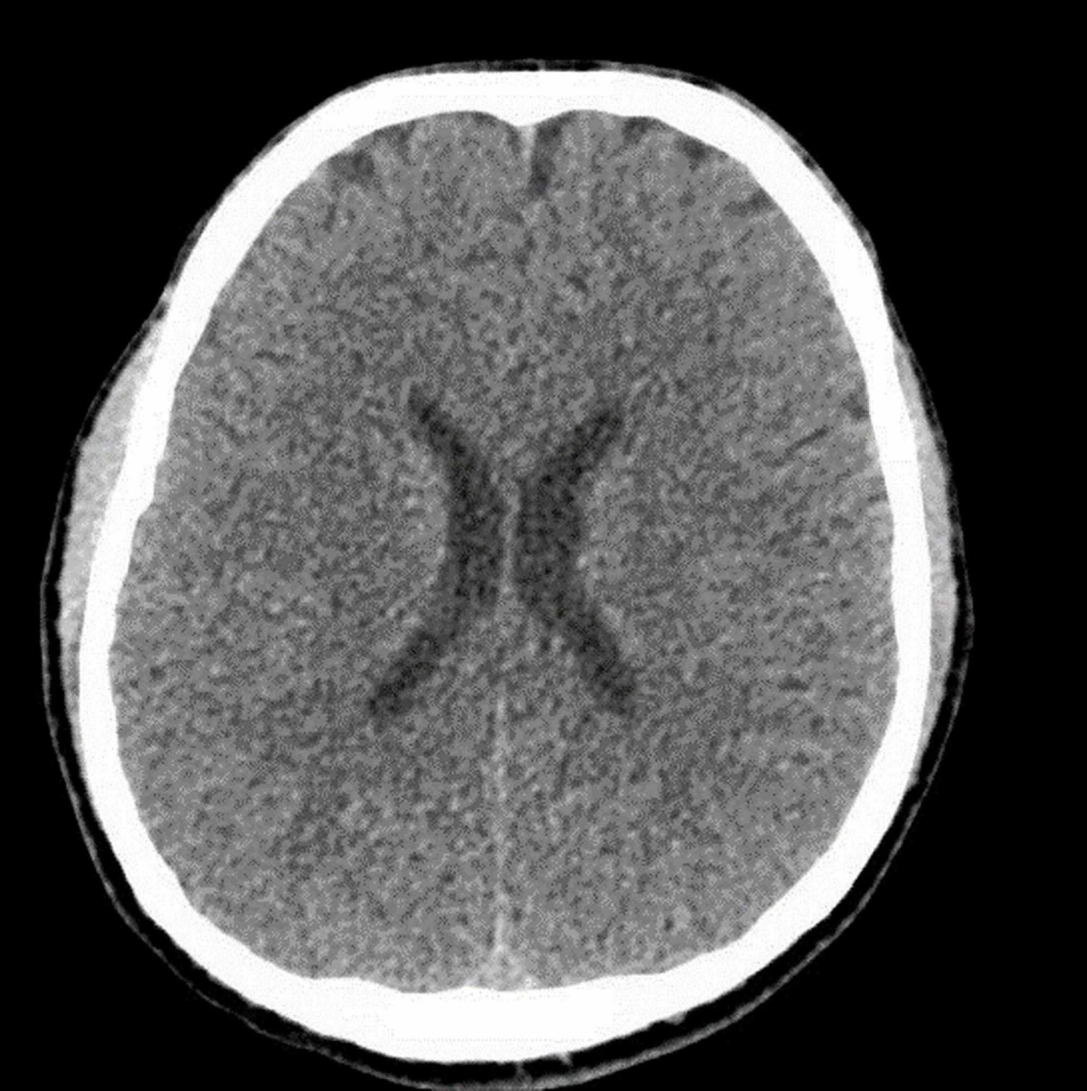
CT scan of the brain: unremarkable for any acute insult.

**Figure 2:**
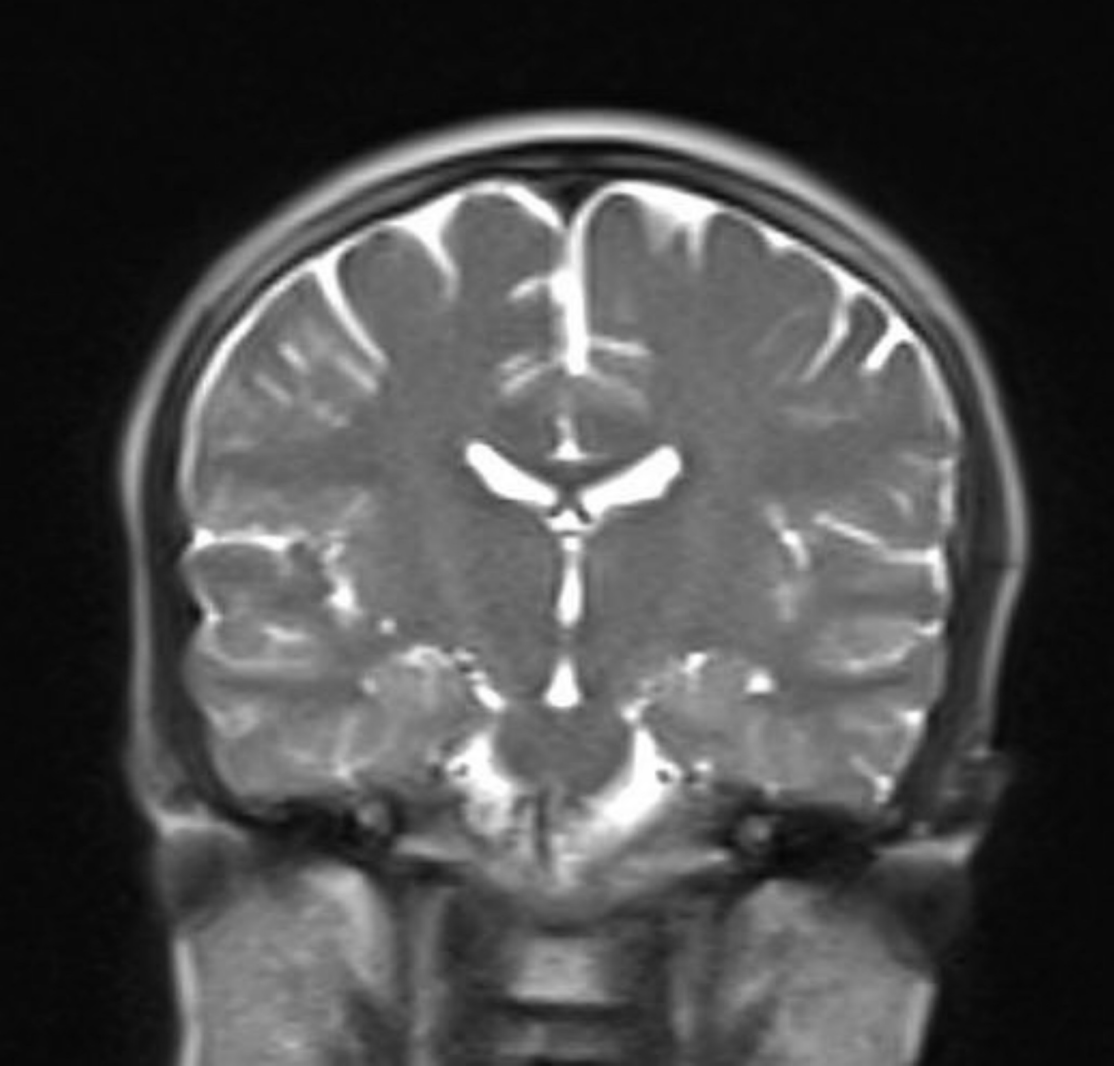
MRI of the brain: unremarkable study.

The neurologist was consulted, and an urgent CT scan (Figure 1) and MRI of the brain (Figure 2) were arranged. It was unremarkable for any acute or chronic pathological findings.

**Figure 3:**
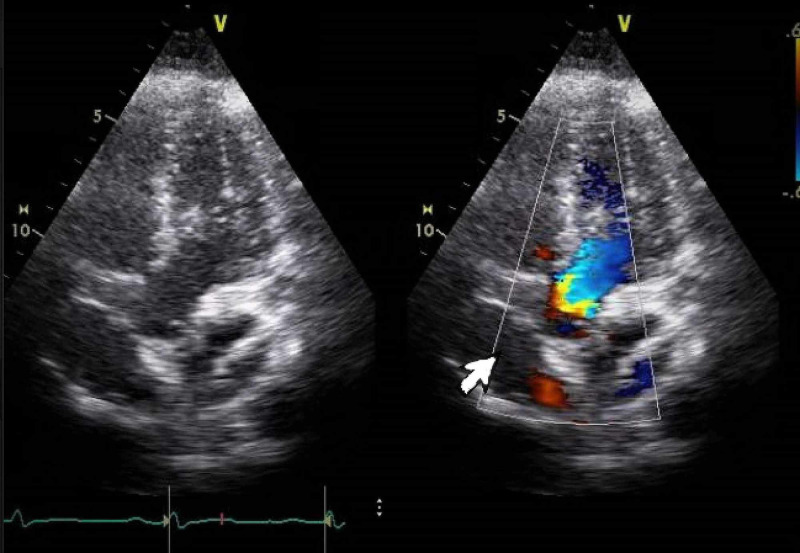
Transthoracic echocardiography with colored Doppler flow: white arrow shows patent foramen ovale.

Upper and lower extremities venous Doppler ultrasound studies ruled out deep venous thrombosis. Echocardiography revealed a large atrial septal aneurysm (ASA) and a small PFO with shunting, accentuated on the Valsalva maneuver, as shown in Figure 3. Holter monitoring for 48 hours did not show any abnormal heart rhythm. As our patient was young and healthy, he was investigated for thrombophilia disorders including Factor V Leiden mutation, Protein C and protein S deficiency, lupus screen, essential thrombocytosis with JAK-STAT mutation, and homocystinuria. Flow cytometry for paroxysmal nocturnal hemoglobinuria (PNH) was negative. Autoimmune disease and mixed connective tissue disease workup were also unremarkable. syphilis, HIV, and hepatitis screenings were negative as well.

Based on thorough investigations, it was concluded that BRAO has resulted from cardioembolic phenomena due to concurrent PFO and ASA. The cardiologist service was consulted, and the patient was commenced on aspirin. His vision started to improve during his hospital stay, and there were no further complications. He was discharged with outpatient follow-ups with an interventional cardiologist for possible corrective surgery of PFO. He was followed for one year. During his follow-up, he traveled abroad for a second opinion and was started on warfarin for a short duration. After he arrived back, he was seen by the cardiologist and ophthalmologists in the outpatient department. There was no indication to keep the patient on warfarin; therefore, it was stopped and aspirin was continued. He was having a large ASA and did not fit the criteria of corrective surgery for PFO. Aspirin was prescribed for lifelong with regular follow-up with the cardiologist as an outpatient, and the patient remained stable.

## Discussion

CRAO is rare in patients younger than 25 years, but if present, it is mostly attributed to underlying systemic diseases such as coagulopathies, collagen vascular diseases, or autoimmune diseases. In the elderly, the underlying etiology is mostly atheromatous plaque in the carotid arteries or valvular heart disease. Cardioembolic phenomena can occur in both young and elderly populations [1,2,4]. PFO is a physiological variant of persistent communication between the left and right atrium in fetal life. In a study by Meier and Lock, 45% of the cryptogenic strokes were associated with PFO, and up to 23% of the patients presented with CRAO [2]. However, in younger patients younger than 45 years with CRAO associated with cardiac defect may benefit from correctional surgery [3,4,7]. Around 40% of acute ischemic strokes are reportedly labeled as cryptogenic or of undetermined etiology. This number is significant in the younger population, mainly due to inadequate diagnostic workup, multiple causes, or an unrecognized etiology contributing to this statistic [1,3].

Upon the literature review, Nakagawa et al. presented a case of bilateral CRAO in a patient with PFO and deep venous thrombosis [2]. Clifford et al. described CRAO and ischemic optic neuropathy in a young patient with PFO [4]. Gabrielian et al. reported CRAO in a healthy 17-year-old male patient with sudden, painless vision loss. Although routine diagnostic workup, including transthoracic echocardiography (TTE), was negative, TEE could establish a PFO [8]. In another case report, Sheth et al. described the case of an 18-year-old man with a hemi-retinal artery occlusion presented with sudden onset visual loss on the left side. Subsequently, TEE could demonstrate a PFO, which was later closed surgically [9]. Mohamed et al. reported the case of a 24-year-old man with a history of migraine and visual auras from the age of 16 years who presented with sudden-onset painless scotoma in his right eye. He was diagnosed based on localized retinal artery obstruction and cotton wool spots. Comprehensive physical examination, medical history, and initial laboratory tests were unremarkable. A contrast echocardiogram showed right-to-left shunting at the atrial level and a PFO [10].

Here we share our experience of cardioembolic phenomena associated with ASA and PFO in a healthy young man leading to BRAO. Our patient’s history was remarkable for engagement in swimming on that day. He later relaxed to watch a football match on TV and had acute onset of blurring of vision. He was diagnosed as a case of BRAO on an ophthalmoscope examination. An exertional activity might be the reason for his clinical presentation causing paradoxical embolism and BRAO. PFO is usually asymptomatic; it should be considered among the possible causes of BRAO/CRAO [1]. Only a few numbers of reported cases described BRAO in association with PFO, which is rarer than CRAO and retinal artery occlusion due to PFO, as described by Greven et al. and Shannon et al. [5,6]. We investigated thoroughly with brain CT scan, echocardiography, thrombophilia workup, and autoimmune screening along with neurologist and ophthalmologist consultations to rule out all the possible causes of BRAO in our patient, as mentioned in various reported cases in the literature [11-13]. His workup revealed PFO with ASA on TTE. The choice of modality is trans-esophageal echocardiography if TTE is not showing PFO and there is a high suspicion of cardiac abnormality [3]. However, in our case, there was concurrent PFO and ASA as an underlying etiology of BRAO, leading to cardioembolic phenomena, thus making treatment even more complicated. Such presentation has not been reported as per our knowledge; however, case reports of BRAO and associated PFO only are in the literature [5,6,14,15]. Current guidelines advise for corrective surgery in a patient with PFO leading to cryptogenic stroke [7]. However, if there is a concurrent pathology such as ASA, as in our case, then a careful decision is made. Even after corrective surgery, ASA is itself an independent risk of stroke, and anticoagulation may be warranted for lifelong [3].

## Conclusions

We aim to highlight the association and management of BRAO in a healthy young man due to concurrent PFO and ASA, which is not much reported in the literature. We have to exclude other possible differential diagnoses as well before considering PFO as a cause. Echocardiography, specifically trans-esophageal, is the modality of choice to diagnose. Early diagnosis and close follow-up are warranted along with the involvement of different departments such as neurology, cardiology, and ophthalmology to prevent a grave complication of loss of vision.
